# *N*-representability of the Jastrow wave function pair density of the lowest-order

**DOI:** 10.1038/s41598-017-07454-8

**Published:** 2017-08-08

**Authors:** Katsuhiko Higuchi, Masahiko Higuchi

**Affiliations:** 1 Graduate School of Advanced Sciences of Matter, Hiroshima University, Higashi-Hiroshima, 739-8527 Japan; 2 Department of Physics, Faculty of Science, Shinshu University, Matsumoto, 390-8621 Japan

## Abstract

Conditions for the *N*-representability of the pair density (PD) are needed for the development of the PD functional theory. We derive sufficient conditions for the *N*-representability of the PD that is calculated from the Jastrow wave function within the lowest order. These conditions are used as the constraints on the correlation function of the Jastrow wave function. A concrete procedure to search the suitable correlation function is also presented.

## Introduction

The pair density (PD), which is defined as the diagonal element of the second-order reduced density matrix, has more information about the electron correlation than the electron density^1–4^. Therefore, the PD functional theory has been expected to be one of the promising schemes beyond the density functional theory^5–28^. We have proposed the PD functional theory that yields the best PD within the set of the Jastrow wave function PDs of the lowest-order (LO-Jastrow PDs)^17^. The search region for the ground-state PD is substantially extended as compared with the previous theory^18, 19^. On the other hand, however, there remains a significant problem related to the *N*-representability of the LO-Jastrow PDs^17^. Concerning the *N*-representability of the PD or the second-order reduced density matrix, there are a lot of works^29–39^. But unfortunately, the necessary and sufficient conditions for the *N*-representability of the PD are not yet known in a practical form.

Let us revisit the problem here. We shall consider an *N*_0_-electron system. The Jastrow wave function is given by

1 $${{\rm{\Psi }}}_{J}({{x}}_{1},\cdot \cdot \cdot ,{{x}}_{{N}_{0}})=\frac{1}{\sqrt{{A}_{{N}_{0}}}}\prod _{1\le i < j\le {N}_{0}}f(|{{\bf{r}}}_{i}-{{\bf{r}}}_{j}|)\,{{\rm{\Phi }}}_{SSD}({{x}}_{1},\cdot \cdot \cdot ,{{x}}_{{N}_{0}}),$$

where *x*_*i*_ denotes the coordinates including the spatial coordinate **r**_i_ and spin coordinate *η*_*i*_, and where ${A}_{{N}_{0}}$, $f(|{{\bf{r}}}_{i}-{{\bf{r}}}_{j}|)$ and ${{\rm{\Phi }}}_{SSD}({{x}}_{1},\cdot \cdot \cdot ,{{x}}_{{N}_{0}})$ are the normalization constant, correlation function and single Slater determinant (SSD), respectively. The LO-Jastrow PD is given by^17, 40, 41^

2 $${\gamma }_{LO}^{\mathrm{(2)}}({\bf{rr}}^{\prime} ;{\bf{rr}}^{\prime} )={|f(|{{\bf{r}}}_{i}-{{\bf{r}}}_{j}|)|}^{2}{\gamma }_{SSD}^{\mathrm{(2)}}{({\bf{rr}}^{\prime} ;{\bf{rr}}^{\prime} )}_{N={N}_{0}},$$

where ${\gamma }_{SSD}^{\mathrm{(2)}}{({\bf{rr}}^{\prime} ;{\bf{rr}}^{\prime} )}_{N={N}_{0}}$ is the PD calculated from the SSD. In the preceding paper^17^, we have confirmed that Eq. (2) meets four kinds of necessary conditions for the *N*-representability of the PD, and may become “approximately *N*-representable”^42–47^. However, the possibility of it being *N*-representable has not been discussed^17^. This is an arguable problem that is concerned with whether the reproduced PD is physically reasonable or not.

The aim of this paper is to discuss the *N*-representability of Eq. (2) and to show the way to search the suitable correlation function $f(|{{\bf{r}}}_{i}-{{\bf{r}}}_{j}|)$. The organization of this paper is as follows. For the convenience of the subsequent discussions, we first examine the properties of the LO-Jastrow PD in the next section. Then, the sufficient conditions for the *N*-representability of Eq. (2), which are imposed on the correlation function, will be derived recursively. Next, concrete steps for searching the correlation function that meets these conditions are discussed. Finally, concluding remarks are given in the last section.

## Results

### Properties of the LO-Jastrow PD

In this section, we shall discuss the properties of the LO-Jastrow PD. To this aim, the properties of PDs that are calculated from SSDs are investigated. The cofactor expansion of ${{\rm{\Phi }}}_{SSD}({{x}}_{1},\cdot \cdot \cdot ,{{x}}_{{N}_{0}})$ along the *N*_0_th row leads to

3 $$\begin{array}{rcl}{{\rm{\Phi }}}_{SSD}({{x}}_{1},\cdot \cdot \cdot ,{{x}}_{{N}_{0}}) & = & \frac{1}{\sqrt{{N}_{0}}}\{{(-\mathrm{1)}}^{{N}_{0}+1}{\varphi }_{\lambda }({{x}}_{{N}_{0}}){{\rm{\Phi }}}_{SSD}^{1}({{x}}_{1},\ldots ,{{x}}_{{N}_{0}-1})\\  &  & +{(-\mathrm{1)}}^{{N}_{0}+2}{\varphi }_{\mu }({{x}}_{{N}_{0}}){{\rm{\Phi }}}_{SSD}^{2}({{x}}_{1},\ldots ,{{x}}_{{N}_{0}-1})\\  &  & +\ldots \\  &  & +{(-\mathrm{1)}}^{2{N}_{0}}{\varphi }_{\xi }({x}_{{N}_{0}}){{\rm{\Phi }}}_{SSD}^{{N}_{0}}({x}_{1},\ldots ,{x}_{{N}_{0}-1})\},\end{array}$$

where ${\varphi }_{\lambda }({x}_{{N}_{0}})$, ${\varphi }_{\mu }({x}_{{N}_{0}})$ … and ${\varphi }_{\xi }(x{N}_{0})$, are the constituent spin orbitals of ${{\rm{\Phi }}}_{SSD}({x}_{1},\cdot \cdot \cdot ,{x}_{{N}_{0}})$. In what follows, suppose that these spin orbitals are given as the solutions of simultaneous equations of previous work^17^, and therefore they are orthonormal to each other. ${{\rm{\Phi }}}_{SSD}^{i}({x}_{1},\cdot \cdot \cdot ,{x}_{{N}_{0}-1})$ (1 ≤ *i* ≤ *N*_0_) in Eq. (3) denote (*N*_0_ − 1) -electron SSDs that are defined as the minor determinants multiplied by $\mathrm{1/}\sqrt{({N}_{0}-\mathrm{1)!}}$. The PD that is calculated from Eq. (3) is given by

4 $${\gamma }_{SSD}^{\mathrm{(2)}}{({\bf{rr}}^{\prime} ;{\bf{rr}}^{\prime} )}_{N={N}_{0}}=\frac{1}{{N}_{0}-2}\sum _{i\mathrm{=1}}^{{N}_{0}}{\gamma }_{SSD}^{\mathrm{(2)}}{({\bf{rr}}^{\prime} ;{\bf{rr}}^{\prime} )}_{N={N}_{0}-1}^{i},$$

where ${\gamma }_{SSD}^{\mathrm{(2)}}{({\bf{rr}}^{\prime} ;{\bf{rr}}^{\prime} )}_{N={N}_{0}-1}^{i}$ is the PD calculated from ${{\rm{\Phi }}}_{SSD}^{i}({{x}}_{1},\cdot \cdot \cdot ,{{x}}_{{N}_{0}-1})$.

Likewise, the cofactor expansion of each ${{\rm{\Phi }}}_{SSD}^{i}({{x}}_{1},\cdot \cdot \cdot ,{{x}}_{{N}_{0}-1})$ yields (*N*_0_ − 2)-electron SSDs, which are denoted by ${{\rm{\Phi }}}_{SSD}^{ij}({{x}}_{1},\cdot \cdot \cdot ,{{x}}_{{N}_{0}-2})$ (1 ≤ *j *≤ *N*_0_ − 1). Then, each ${\gamma }_{SSD}^{\mathrm{(2)}}{({\bf{rr}}^{\prime} ;{\bf{rr}}^{\prime} )}_{N={N}_{0}-1}^{i}$ is given by $\sum _{j=1}^{{N}_{0}-1}{\gamma }_{SSD}^{\mathrm{(2)}}$ ${({\bf{rr}}^{\prime} ;{\bf{rr}}^{\prime} )}_{N={N}_{0}-2}^{ij}/({N}_{0}-\mathrm{3)}$, where ${\gamma }_{SSD}^{\mathrm{(2)}}{({\bf{rr}}^{\prime} ;{\bf{rr}}^{\prime} )}_{N={N}_{0}-2}^{ij}$ is the PD calculated from ${{\rm{\Phi }}}_{SSD}^{ij}({{x}}_{1},\cdot \cdot \cdot ,{{x}}_{{N}_{0}-2})$. Thus, ${\gamma }_{SSD}^{\mathrm{(2)}}{({\bf{rr}}^{\prime} ;{\bf{rr}}^{\prime} )}_{N={N}_{0}}$ can be expressed by the sum of ${\gamma }_{SSD}^{\mathrm{(2)}}{({\bf{rr}}^{\prime} ;{\bf{rr}}^{\prime} )}_{N={N}_{0}-2}^{ij}$. By the repetition of this procedure, we arrive at

5 $${\gamma }_{SSD}^{\mathrm{(2)}}{({\bf{rr}}^{\prime} ;{\bf{rr}}^{\prime} )}_{N={N}_{0}}=\frac{1}{({N}_{0}-\mathrm{2)!}}\sum _{i\mathrm{=1}}^{{N}_{0}}\sum _{j\mathrm{=1}}^{{N}_{0}-1}\cdot \cdot \cdot \sum _{p\mathrm{=1}}^{4}\sum _{q\mathrm{=1}}^{3}{\gamma }_{SSD}^{\mathrm{(2)}}{({\bf{rr}}^{\prime} ;{\bf{rr}}^{\prime} )}_{N\mathrm{=2}}^{ij\cdot \cdot \cdot pq},$$

where ${\gamma }_{SSD}^{\mathrm{(2)}}{({\bf{rr}}^{\prime} ;{\bf{rr}}^{\prime} )}_{N\mathrm{=2}}^{ij\cdot \cdot \cdot pq}$ $\mathrm{(1}\le i\le {N}_{0},\,1\le j\le {N}_{0}-\mathrm{1,}\cdot \cdot \cdot ,1\le p\le \mathrm{4,1}\le q\le \mathrm{3)}$ are PDs calculated from the two-electron SSDs. These two-electron SSDs, which are denoted by ${{\rm{\Phi }}}_{SSD}^{ij\cdot \cdot \cdot pq}({{x}}_{1},{{x}}_{2})$, are obtained by the above-mentioned successive cofactor expansions.

Multiplying both sides of Eq. (5) by ${|f(|{\bf{r}}-{\bf{r}}{\boldsymbol{^{\prime} }}|)|}^{2}$, we finally get

6 $${|f(|{\bf{r}}-{\bf{r}}{\boldsymbol{^{\prime} }}|)|}^{2}{\gamma }_{SSD}^{\mathrm{(2)}}{({\bf{r}}{\bf{r}}{\boldsymbol{^{\prime} }};{\bf{r}}{\bf{r}}{\boldsymbol{^{\prime} }})}_{N={N}_{0}}=\frac{1}{({N}_{0}-\mathrm{2)!}}\sum _{i=1}^{{N}_{0}}\sum _{j=1}^{{N}_{0}-1}\cdot \cdot \cdot \sum _{p=1}^{4}\sum _{q=1}^{3}|f(|{\bf{r}}-{\bf{r}}{\boldsymbol{^{\prime} }}|)|{}^{2}{\gamma }_{SSD}^{\mathrm{(2)}}{({\bf{r}}{\bf{r}}{\boldsymbol{^{\prime} }};{\bf{r}}{\bf{r}}{\boldsymbol{^{\prime} }})}_{N=2}^{ij\cdot \cdot \cdot pq}\mathrm{.}$$

This relation is the starting point to examine the *N*-representability of the LO-Jastrow PD.

### Sufficient conditions for the *N*-representablity of the LO-Jastrow PD

We shall start with considering the *N*-representability of ${|f(|{\bf{r}}-{\bf{r}}{\boldsymbol{^{\prime} }}|)|}^{2}{\gamma }_{SSD}^{\mathrm{(2)}}{({\bf{r}}{\bf{r}}{\boldsymbol{^{\prime} }};{\bf{r}}{\bf{r}}{\boldsymbol{^{\prime} }})}_{N\mathrm{=2}}^{ij\cdot \cdot \cdot pq}$ that appears in the right-hand side of Eq. (6). Suppose that the two-electron wave function that yields ${|f(|{\bf{r}}-{\bf{r}}{\boldsymbol{^{\prime} }}|)|}^{2}{\gamma }_{SSD}^{\mathrm{(2)}}{({\bf{r}}{\bf{r}}{\boldsymbol{^{\prime} }};{\bf{r}}{\bf{r}}{\boldsymbol{^{\prime} }})}_{N\mathrm{=2}}^{ij\cdot \cdot \cdot pq}$ is given by the following Jastrow wave function;

7 $${{\rm{\Psi }}}_{N\mathrm{=2}}^{ij\cdot \cdot \cdot pq}({{x}}_{1},\,{{x}}_{2})=\frac{1}{\sqrt{{A}_{2}^{ij\cdot \cdot \cdot pq}}}f(|{{\bf{r}}}_{1}-{{\bf{r}}}_{2}|){{\rm{\Phi }}}_{SSD}^{ij\cdot \cdot \cdot pq}({{x}}_{1},{{x}}_{2}),$$

where ${A}_{2}^{ij\cdot \cdot \cdot pq}$ is the normalization constant. From Eq. (7), the PD is calculated as ${|f(|{\bf{r}}-{\bf{r}}^{\prime} |)|}^{2}{\gamma }_{SSD}^{\mathrm{(2)}}{({\bf{rr}}^{\prime} ;{\bf{rr}}^{\prime} )}_{N\mathrm{=2}}^{ij\cdot \cdot \cdot pq}/{A}_{2}^{ij\cdot \cdot \cdot pq}$. Therefore, we get ${A}_{2}^{ij\cdot \cdot \cdot pq}=1$ as the sufficient condition for the *N*-representability of ${|f(|{\bf{r}}-{\bf{r}}^{\prime} |)|}^{2}{\gamma }_{SSD}^{\mathrm{(2)}}{({\bf{rr}}^{\prime} ;{\bf{rr}}^{\prime} )}_{N\mathrm{=2}}^{ij\cdot \cdot \cdot pq}$. Hereafter, we assume that such conditions hold for all values of $i,j,\cdot \cdot \cdot ,p$ and *q*, i.e.,

8 $${A}_{2}^{ij\cdot \cdot \cdot pq}=1\,{\rm{for}}\,{\rm{all}}\,{\rm{values}}\,{\rm{of}}\,i,j,\cdot \cdot \cdot ,p\,{\rm{and}}\,q\mathrm{.}$$

Note that the normalization constant ${A}_{2}^{ij\cdot \cdot \cdot pq}$ is determined by both $f(|{{\bf{r}}}_{1}-{{\bf{r}}}_{2}|)$ and ${{\rm{\Phi }}}_{SSD}^{ij\cdot \cdot \cdot pq}({x}_{1},{x}_{2})$ ^40, 41^. Therefore, if they are given, we can calculate ${A}_{2}^{ij\cdot \cdot \cdot pq}$, and check whether the conditions Eq. (8) are satisfied or not. As mentioned later, the conditions Eq. (8) are the parts of sufficient conditions for the *N*-representability of Eq. (2).

Under the conditions Eq. (8), we have

9 $${|f(|{\bf{r}}-{\bf{r}}^{\prime} |)|}^{2}{\gamma }_{SSD}^{\mathrm{(2)}}{({\bf{rr}}^{\prime} ;{\bf{rr}}^{\prime} )}_{N=2}^{ij\cdot \cdot \cdot pq}=\langle {{\rm{\Psi }}}_{N\mathrm{=2}}^{ij\cdot \cdot \cdot pq}|{\hat{\gamma }}^{\mathrm{(2)}}{({\bf{rr}}^{\prime} ;{\bf{rr}}^{\prime} )}_{N=2}|{{\rm{\Psi }}}_{N=2}^{ij\cdot \cdot \cdot pq}\rangle ,$$

where ${\hat{\gamma }}^{\mathrm{(2)}}{({\bf{rr}}^{\prime} ;{\bf{rr}}^{\prime} )}_{N=n}$ denotes the PD operator for an *n*-electron system. Substituting Eq. (9) into Eq. (6) and rearranging, we get

10 $$\begin{array}{c}{|f(|{\rm{r}}-r\text{'}|)|}^{2}{\gamma }_{SSD}^{\mathrm{(2)}}{({\bf{rr}}^{\prime} ;{\bf{rr}}^{\prime} )}_{N={N}_{0}}\\ \quad =\sum _{i\mathrm{=1}}^{{N}_{0}}[\frac{1}{{N}_{0}-2}\sum _{j\mathrm{=1}}^{{N}_{0}-1}[\frac{1}{{N}_{0}-3}\sum _{k\mathrm{=1}}^{{N}_{0}-2}[\cdot \cdot \cdot \frac{1}{3}\sum _{o\mathrm{=1}}^{5}[\frac{1}{2}\sum _{p\mathrm{=1}}^{4}[\frac{1}{1}\sum _{q\mathrm{=1}}^{3}\langle {{\rm{\Psi }}}_{N\mathrm{=2}}^{ijk\cdot \cdot \cdot opq}|{\hat{\gamma }}^{\mathrm{(2)}}{({\bf{rr}}^{\prime} ;{\bf{rr}}^{\prime} )}_{N\mathrm{=2}}|{{\rm{\Psi }}}_{N\mathrm{=2}}^{ijk\cdot \cdot \cdot opq}\rangle ]]]]]\end{array}$$

It should be noticed that the right-hand side of Eq. (10) has a characteristic form. Concerning the *N*-representability of this form, the following theorem holds:

*Theorem*. If there exists the set of single-valued, continuous, smooth and finite functions {*a*_*α*_(*x*_*n* + 1_)} (1 ≤ *α* ≤ *n* + 1) that satisfy the conditions;

11 $$\int {a}_{\alpha }^{\ast }({{x}}_{n+1}){a}_{\alpha \text{'}}({{x}}_{n+1})d\,{x}_{n+1}=\frac{1}{n+1}{\delta }_{\alpha \alpha \text{'}},$$

12 $$\sigma \sum _{\alpha =1}^{n+1}{a}_{\alpha }({{x}}_{n+1}){{\rm{\Psi }}}_{N=n}^{\alpha }({{x}}_{1},\cdot \cdot \cdot ,{{x}}_{n})={(-\mathrm{1)}}^{\bar{\sigma }}\sum _{\alpha =1}^{n+1}{a}_{\alpha }({{x}}_{n+1}){{\rm{\Psi }}}_{N=n}^{\alpha }({{x}}_{1},\cdot \cdot \cdot ,{{x}}_{n}),$$

then the following equations hold:

13 $$\frac{1}{n-1}\sum _{\alpha \mathrm{=1}}^{n+1}\langle {{\rm{\Psi }}}_{N=n}^{\alpha }|{\hat{\gamma }}^{\mathrm{(2)}}{({\bf{rr}}^{\prime} ;{\bf{rr}}^{\prime} )}_{N=n}|{{\rm{\Psi }}}_{N=n}^{\alpha }\rangle =\langle {{\rm{\Psi }}}_{N=n+1}|{\hat{\gamma }}^{\mathrm{(2)}}{({\bf{rr}}^{\prime} ;{\bf{rr}}^{\prime} )}_{N=n+1}|{{\rm{\Psi }}}_{N=n+1}\rangle $$

with

14 $${{\rm{\Psi }}}_{N=n+1}({{x}}_{1},\cdot \cdot \cdot ,{{x}}_{n+1})=\sum _{\alpha \mathrm{=1}}^{n+1}{a}_{\alpha }({x}_{n+1}){{\rm{\Psi }}}_{N=n}^{\alpha }({{x}}_{1},\cdot \cdot \cdot ,{{x}}_{n}\mathrm{).}$$

Here ${{\rm{\Psi }}}_{N=n}^{\alpha }({{x}}_{1},\,\cdot \cdot \cdot ,\,{{x}}_{n})$ (1 ≤ *α* ≤ *n* + 1) denote the *n*-electron wave functions, and *σ* is a permutation operator upon the electron coordinates, and $\bar{\sigma }$ is the number of interchanges in *σ*.

*Proof*. The left-hand side of Eq. (13) seems to be related to the average of ${\hat{\gamma }}^{\mathrm{(2)}}{({\bf{rr}}^{\prime} ;{\bf{rr}}^{\prime} )}_{N=n}$ with respect to a density matrix for a mixed state. Indeed, if the density matrix for the mixed state is given by

15 $${\hat{\rho }}_{n}=\sum _{\alpha \mathrm{=1}}^{n+1}\frac{1}{n+1}|{{\rm{\Psi }}}_{N=n}^{\alpha }\rangle \langle {{\rm{\Psi }}}_{N=n}^{\alpha }|,$$

then the average of ${\hat{\gamma }}^{\mathrm{(2)}}{({\bf{rr}}^{\prime} ;{\bf{rr}}^{\prime} )}_{N=n}$ is calculated as

16 $${\rm{Tr}}\,[{\hat{\rho }}_{n}{\hat{\gamma }}^{\mathrm{(2)}}{({\bf{rr}}^{\prime} ;{\bf{rr}}^{\prime} )}_{N=n}]=\frac{1}{n+1}\sum _{\alpha \mathrm{=1}}^{n+1}\langle {{\rm{\Psi }}}_{N=n}^{\alpha }|{\hat{\gamma }}^{\mathrm{(2)}}{({\bf{rr}}^{\prime} ;{\bf{rr}}^{\prime} )}_{N=n}|{{\rm{\Psi }}}_{N=n}^{\alpha }\rangle \mathrm{.}$$

By using Eq. (16), the left-hand side of Eq. (13) is rewritten as $\{(n+\mathrm{1)/(}n-\mathrm{1)}\}\,{\rm{Tr}}\,[{\hat{\rho }}_{n}{\hat{\gamma }}^{\mathrm{(2)}}{({\bf{rr}}^{\prime} ;{\bf{rr}}^{\prime} )}_{N=n}]$.

On the other hand, it is expected that the average ${\rm{Tr}}\,[{\hat{\rho }}_{n}{\hat{\gamma }}^{\mathrm{(2)}}{({\bf{rr}}^{\prime} ;{\bf{rr}}^{\prime} )}_{N=n}]$ may be given as the expectation value of ${\hat{\gamma }}^{\mathrm{(2)}}{({\bf{rr}}^{\prime} ;{\bf{rr}}^{\prime} )}_{N=n}$ with respect to a pure state for the whole system that includes the *n*-electron system as a subsystem^48, 49^. We shall take an (*n *+ 1)-electron system as the whole system, and suppose that the wave function for such the (*n* + 1)-electron system is given by Eq. (14) with Eq. (11). Then we indeed get

17 $$\langle {{\rm{\Psi }}}_{n+1}|{\hat{\gamma }}^{\mathrm{(2)}}{({\bf{rr}}^{\prime} ;{\bf{rr}}^{\prime} )}_{N=n}|{{\rm{\Psi }}}_{n+1}\rangle ={\rm{Tr}}\,[{\hat{\rho }}_{n}{\hat{\gamma }}^{\mathrm{(2)}}{({\bf{rr}}^{\prime} ;{\bf{rr}}^{\prime} )}_{N=n}\mathrm{].}$$

Furthermore, if ${{\rm{\Psi }}}_{N=n+1}({x}_{1},\cdot \cdot \cdot ,{x}_{n+1})$ is antisymmetric, i.e., if Eq. (12) holds, then the expectation value of ${\hat{\gamma }}^{\mathrm{(2)}}{({\bf{rr}}^{\prime} ;{\bf{rr}}^{\prime} )}_{N=n+1}$ with respect to ${{\rm{\Psi }}}_{N=n+1}({x}_{1},\cdot \cdot \cdot ,{x}_{n+1})$ is given by

18 $$\langle {{\rm{\Psi }}}_{n+1}|{\hat{\gamma }}^{\mathrm{(2)}}({\bf{rr}}^{\prime} ;{\bf{rr}}{^{\prime} }_{N=n+1}|{{\rm{\Psi }}}_{n+1}\rangle =\frac{n+1}{n-1}\langle {{\rm{\Psi }}}_{n+1}|{\hat{\gamma }}^{\mathrm{(2)}}{({\bf{rr}}^{\prime} ;{\bf{rr}}^{\prime} )}_{N=n}|{{\rm{\Psi }}}_{n+1}\rangle \mathrm{.}$$

Consequently, Eq. (13) immediately follows from Eqs (16), (17) and (18). This means that Eq. (13) holds under the conditions that there exists the set of functions {*a*_*α*_(*x*_*n* + 1_)} (1 ≤ *α* ≤ *n* + 1) that satisfy Eqs (11) and (12). Q.E.D.

First, we apply the above theorem to the term $\frac{1}{1}\sum _{q\mathrm{=1}}^{3}\langle {{\rm{\Psi }}}_{N\mathrm{=2}}^{ijk\cdot \cdot \cdot opq}|{\hat{\gamma }}^{\mathrm{(2)}}{({\bf{rr}}^{\prime} ;{\bf{rr}}^{\prime} )}_{N\mathrm{=2}}|{{\rm{\Psi }}}_{N\mathrm{=2}}^{ijk\cdot \cdot \cdot opq}\rangle$ that appears in Eq. (10). According to the theorem, this term can be rewritten as

19 $$\frac{1}{1}\sum _{q\mathrm{=1}}^{3}\langle {{\rm{\Psi }}}_{N=2}^{ijk\cdot \cdot \cdot opq}|{\hat{\gamma }}^{\mathrm{(2)}}{({\bf{rr}}^{\prime} ;{\bf{rr}}^{\prime} )}_{N=2}|{{\rm{\Psi }}}_{N=2}^{ijk\cdot \cdot \cdot opq}\rangle =\langle {{\rm{\Psi }}}_{N=3}^{ijk\cdot \cdot \cdot op}|{\hat{\gamma }}^{\mathrm{(2)}}{({\bf{rr}}^{\prime} ;{\bf{rr}}^{\prime} )}_{N=3}|{{\rm{\Psi }}}_{N=3}^{ijk\cdot \cdot \cdot op}\rangle $$

with

20 $${{\rm{\Psi }}}_{N\mathrm{=3}}^{ijk\cdot \cdot \cdot op}({{x}}_{1},\,{{x}}_{2},{{x}}_{3})=\sum _{q\mathrm{=1}}^{3}{a}_{q}^{ijk\cdot \cdot \cdot op}({x}_{3}){{\rm{\Psi }}}_{N\mathrm{=2}}^{ijk\cdot \cdot \cdot opq}({{x}}_{1},\,{{x}}_{2}),$$

if there exists the set of functions $\{{a}_{q}^{ijk\cdot \cdot \cdot op}({x}_{3})\}$ (1 ≤ *q* ≤ 3) that satisfy the following conditions:

21 $$\int {a}_{q}^{ijk\cdot \cdot \cdot op}{({{x}}_{3})}^{\ast }{a}_{q^{\prime} }^{ijk\cdot \cdot \cdot op}({{x}}_{3})\,d\,x=\frac{1}{3}{\delta }_{q,q\text{'}},$$

22 $$\sigma \sum _{q\mathrm{=1}}^{3}{a}_{q}^{ijk\cdot \cdot \cdot op}({{x}}_{3}){{\rm{\Psi }}}_{N\mathrm{=2}}^{ijk\cdot \cdot \cdot opq}({{x}}_{1},{{x}}_{2})={(-\mathrm{1)}}^{\bar{\sigma }}\sum _{q\mathrm{=1}}^{3}{a}_{q}^{ijk\cdot \cdot \cdot op}({{x}}_{3}){{\rm{\Psi }}}_{N=2}^{ijk\cdot \cdot \cdot opq}({{x}}_{1},{{x}}_{2}\mathrm{).}$$

In addition to Eq. (8), the existence conditions for $\{{a}_{q}^{ijk\cdot \cdot \cdot op}({{x}}_{3})\}$ (1 ≤ *q* ≤ 3), i.e., Eqs (21) and (22), are also the parts of sufficient conditions for the *N*-representability of Eq. (2). We assume that the set of functions $\{{a}_{q}^{ijk\cdot \cdot \cdot op}({x}_{3})\}$ (1 ≤ *q* ≤ 3) is obtained. Substitution of Eq. (20) into Eq. (10) leads to

23 $$\begin{array}{c}{|f(|{\bf{r}}-{\bf{r}}^{\prime} |)|}^{2}{\gamma }_{SSD}^{\mathrm{(2)}}{({\bf{rr}}^{\prime} ;{\bf{rr}}^{\prime} )}_{N={N}_{0}}\\ =\sum _{i\mathrm{=1}}^{{N}_{0}}[\tfrac{1}{{N}_{0}-2}\sum _{j\mathrm{=1}}^{{N}_{0}-1}[\tfrac{1}{{N}_{0}-3}\sum _{k\mathrm{=1}}^{{N}_{0}-2}[\cdot \cdot \cdot \tfrac{1}{3}\sum _{o\mathrm{=1}}^{5}[\tfrac{1}{2}\sum _{p\mathrm{=1}}^{4}\langle {{\rm{\Psi }}}_{N\mathrm{=3}}^{ijk\cdot \cdot \cdot op}|{\hat{\gamma }}^{\mathrm{(2)}}{({\bf{rr}}^{\prime} ;{\bf{rr}}^{\prime} )}_{N\mathrm{=3}}|{{\rm{\Psi }}}_{N\mathrm{=3}}^{ijk\cdot \cdot \cdot op}\rangle ]]]]\mathrm{.}\end{array}$$

Next, we apply the theorem to the term $\frac{1}{2}\sum _{p\mathrm{=1}}^{4}\langle {{\rm{\Psi }}}_{N\mathrm{=3}}^{ijk\cdot \cdot \cdot op}|{\hat{\gamma }}^{\mathrm{(2)}}{({\bf{rr}}^{\prime} ;{\bf{rr}}^{\prime} )}_{N\mathrm{=3}}|{{\rm{\Psi }}}_{N\mathrm{=3}}^{ijk\cdot \cdot \cdot op}\rangle$ that appears in Eq. (23). Similarly to the above, we obtain

24 $$\frac{1}{2}\sum _{p\mathrm{=1}}^{4}\langle {{\rm{\Psi }}}_{N\mathrm{=3}}^{ijk\cdot \cdot \cdot op}|{\hat{\gamma }}^{\mathrm{(2)}}{({\bf{rr}}^{\prime} ;{\bf{rr}}^{\prime} )}_{N\mathrm{=3}}|{{\rm{\Psi }}}_{N\mathrm{=3}}^{ijk\cdot \cdot \cdot op}\rangle =\langle {{\rm{\Psi }}}_{N\mathrm{=4}}^{ijk\cdot \cdot \cdot o}|{\hat{\gamma }}^{\mathrm{(2)}}{({\bf{rr}}^{\prime} ;{\bf{rr}}^{\prime} )}_{N\mathrm{=4}}|{{\rm{\Psi }}}_{N\mathrm{=4}}^{ijk\cdot \cdot \cdot o}\rangle $$

with

25 $${{\rm{\Psi }}}_{N\mathrm{=4}}^{ijk\cdot \cdot \cdot o}({{x}}_{1},\cdot \cdot \cdot ,{{x}}_{4})=\sum _{p\mathrm{=1}}^{4}{a}_{p}^{ijk\cdot \cdot \cdot o}({x}_{4}){{\rm{\Psi }}}_{N\mathrm{=3}}^{ijk\cdot \cdot \cdot op}({{x}}_{1},\,{{x}}_{2},\,{{x}}_{3}),$$

if there exists the set of functions $\{{a}_{p}^{ijk\cdot \cdot \cdot o}({x}_{4})\}$ (1 ≤ *p* ≤ 4) that satisfy the following conditions:

26 $$\int {a}_{p}^{ijk\cdot \cdot \cdot o}{({{x}}_{4})}^{\ast }{a}_{p^{\prime} }^{ijk\cdot \cdot \cdot o}({{x}}_{4})\,d\,x=\frac{1}{4}{\delta }_{p,p\text{'}},$$

27 $$\sigma \sum _{p\mathrm{=1}}^{4}{a}_{p}^{ijk\cdot \cdot \cdot o}({{x}}_{4}){{\rm{\Psi }}}_{N\mathrm{=3}}^{ijk\cdot \cdot \cdot op}({{x}}_{1},{{x}}_{2},\,{{x}}_{3})={(-\mathrm{1)}}^{\bar{\sigma }}\sum _{p\mathrm{=1}}^{4}{a}_{p}^{ijk\cdot \cdot \cdot o}({{x}}_{4}){{\rm{\Psi }}}_{N\mathrm{=3}}^{ijk\cdot \cdot \cdot op}({{x}}_{1},{{x}}_{2},\,{{x}}_{3}\mathrm{).}$$

The existence conditions for $\{{a}_{p}^{ijk\cdot \cdot \cdot o}({{x}}_{4})\}$ (1 ≤ *p* ≤ 4), i.e., Eqs (26) and (27), are added to the set of sufficient conditions for the *N*-representability of Eq. (2). At this stage, Eq. (8) and the existence conditions for $\{{a}_{q}^{ijk\cdot \cdot \cdot op}({{x}}_{3})\}$ (1 ≤ *q* ≤ 3) and $\{{a}_{p}^{ijk\cdot \cdot \cdot o}({{x}}_{4})\}$ (1 ≤ *p* ≤ 4) belong to the set of sufficient conditions.

Thus the theorem is applied repeatedly, so that further conditions are added to the set of sufficient conditions. Continuing until the conditions for $\{{a}_{i}({{x}}_{{N}_{0}})\}$ (1 ≤ *i* ≤ *N*_0_), we eventually obtain sufficient conditions for the *N*-representability of Eq. (2).

## Discussions

### Concrete steps for constructing the *N*-representable LO-Jastrow PD

In the preceding section, the sufficient conditions for the *N*-representability of the LO-Jastrow PD are derived. In this section, we consider the concrete steps for searching the correlation function that meets these conditions or checking its existence.

1. First, we give a trial form of the correlation function. Using this, simultaneous equations for the *N*_0_-electron system are solved in a self-consistent way^17^.
2. Let us consider the SSD that consists of the resultant spin orbitals for the simultaneous equations. The SSD can generally be expanded using the cofactor. The SSD for the *N*_0_-electron system is expanded along the *N*_0_th row, then we get the *N*_0_ number of SSDs for the (*N*_0_ − 1)-electron system, i.e., ${{\rm{\Phi }}}_{SSD}^{i}({{x}}_{1},\cdot \cdot \cdot ,{{x}}_{{N}_{0}-1})$ (1 ≤ *i* ≤ *N*_0_). Successively, each of the SSDs for the (*N*_0_ − 1)-electron system is expanded along the (*N*_0_ − 1)th row, and then the (*N*_0_ − 1) number of SSDs for the (*N*_0_ − 2)-electron system, i.e., ${{\rm{\Phi }}}_{SSD}^{ij}({{x}}_{1},\cdot \cdot \cdot ,{{x}}_{{N}_{0}-2})$ (1 ≤ *j* ≤ *N*_0_ − 1), can be obtained for each *i*. After that and later, the cofactor expansions are likewise repeated, and we finally arrive at the SSDs for the two-electron system, i.e.,${{\rm{\Phi }}}_{SSD}^{ijk\cdot \cdot \cdot \cdot opq}({{x}}_{1},\,{{x}}_{2})$. The number of two-electron SSDs thus obtained is *N*_0_!/2, since $i,\,j,\,k,\cdot \cdot \cdot \cdot ,\,o,\,p$ and *q* are integers such that 1 ≤ *i* ≤ *N*_0_, 1 ≤ *j* ≤ *N*_0_ − 1, 1 ≤ *k* ≤ *N*_0_ − 2, $\ldots$, 1 ≤ *o* ≤ 5, 1 ≤ p ≤ 4 and 1 ≤ q ≤ 3, respectively.
3. The two-electron SSD ${{\rm{\Phi }}}_{SSD}^{ijk\cdot \cdot \cdot \cdot opq}({{x}}_{1},\,{{x}}_{2})$ and the correlation function determine the normalization constant ${A}_{2}^{ijk\cdot \cdot \cdot \cdot opq}$ ^40, 41^. We check whether all of ${A}_{2}^{ijk\cdot \cdot \cdot \cdot opq}$ are unity or not. If no, we return to the step 1 and change the form of the correlation function. This process proceeds until all of ${A}_{2}^{ijk\cdot \cdot \cdot \cdot opq}$ become unity. Suppose that such the correlation function is found, we get two-electron antisymmetric wave functions ${{\rm{\Psi }}}_{N\mathrm{=2}}^{ijk\cdot \cdot \cdot \cdot opq}({{x}}_{1},\,{{x}}_{2})$ for all cases of $i,\,j,\,k,\,\cdot \cdot \cdot \cdot ,\,o,\,p$ and *q* through Eq. (7).
4. By means of these ${{\rm{\Psi }}}_{N\mathrm{=2}}^{ijk\cdot \cdot \cdot \cdot opq}({{x}}_{1},\,{{x}}_{2})$, three-electron wave functions are defined as Eq. (20). We will check whether there exists the set of $\{{a}_{q}^{ijk\cdot \cdot \cdot \cdot op}({{x}}_{3}),\,q=1,2,3\}$ that are satisfied with Eqs. (21) and (22). Note that the check has to be performed for all cases of $i,\,j,\,k,\,\cdot \cdot \cdot \cdot ,\,o$ and *p*. If suitable $\{{a}_{q}^{ijk\cdot \cdot \cdot \cdot op}({{x}}_{3})\}$ cannot be found, we again return to the step 1 and modify the correlation function. Suppose the suitable $\{{a}_{q}^{ijk\cdot \cdot \cdot \cdot op}({{x}}_{3})\}$ is found in this process, then three-electron antisymmetric wave functions ${{\rm{\Psi }}}_{N\mathrm{=3}}^{ijk\cdot \cdot \cdot \cdot op}({{x}}_{1},\,{{x}}_{2},{{x}}_{3})$ for all cases of $i,j,k,\ldots ,o$ and *p* can be constructed from Eq. (20).
5. We successively proceed the case of four-electron wave functions that are defined as Eq. (25). In a similar way to the step 4, we check whether the set of $\{{a}_{p}^{ijk\cdot \cdot \cdot \cdot o}({{x}}_{4}),\,p=1,2,3,4\}$ meets the conditions of Eqs. (26) and (27). If no, we restart from the step 1 with the modified correlation function. Suppose that the suitable $\{{a}_{p}^{ijk\ldots o}({{x}}_{4})\}$ are found for all cases of $i,\,j,\,k,\ldots$ and *o*, the four-electron antisymmetric wave functions ${{\rm{\Psi }}}_{N=4}^{ijk\ldots o}({{x}}_{1},\,\ldots ,{{x}}_{4})$ can be obtained from Eq. (25).
6. Likewise, we further proceed the problem constructing the antisymmetric wave functions for more-electron systems. We search the set of $\{{a}_{\alpha }({{x}}_{n+1}),\alpha =1,2,\ldots ,n+1\}$ that are satisfied with Eqs. (11) and (12), together with modifying the correlation function. If we successfully find the correlation function that meets the conditions (11) and (12) for any *n*(≤*N*_0_ − 1), the LO-Jastrow PD of the *N*_0_-electron system becomes *N*-representable.

As easily inferred, the above steps are feasible only for the small-electron systems from the practical viewpoint. However, it should be noted that the correlation function that makes the LO-Jastrow PD *N*-representable may, in principle, be found along the above steps, though there is a possibility that the suitable correlation function may not exist in some system.

### Other possibilities to obtain antisymmetric wave functions

In the above-mentioned concrete steps, antisymmetric (n + 1)-electrons wave functions are built up from given antisymmetric n-electrons wave functions via Eq. (14). In this subsection, we show that there are other possibilities to obtain antisymmetric (n+1)-electrons wave functions without using Eq. (14).

Instead of Eq. (14), we can use the following expression for ${{\rm{\Psi }}}_{N=n+1}({{x}}_{1},\cdot \cdot \cdot ,{{x}}_{n+1})$;

28 $${{\rm{\Psi }}}_{N=n+1}({{x}}_{1},\cdot \cdot \cdot ,{{x}}_{n+1})=\sum _{\alpha \mathrm{=1}}^{n+1}\prod _{i\mathrm{=1}}^{n}{a}_{\alpha }({{x}}_{i}){b}_{\alpha }({{x}}_{n+1}){{\rm{\Psi }}}_{N=n}^{\alpha }({{x}}_{1},\cdot \cdot \cdot ,{{x}}_{n}),$$

where *a*_*α*_(*x*_*i*_) and *b*_*α*_(*x*_*n* + 1_) denotes functions that should be determined. If these functions satisfy the following conditions;

29 $${|{a}_{\alpha }({x})|}^{2}\mathrm{=1,}$$

30 $$\int {b}_{\alpha \text{'}}{({{x}}_{n+1})}^{\ast }{b}_{\alpha }({{x}}_{n+1})d{{x}}_{n+1}=\frac{1}{n+1}{\delta }_{\alpha ,\alpha \text{'}},$$

31 $$\sigma \sum _{\alpha \mathrm{=1}}^{n+1}\prod _{i\mathrm{=1}}^{n}{a}_{\alpha }({{x}}_{i}){b}_{\alpha }({{x}}_{n+1}){{\rm{\Psi }}}_{N=n}^{\alpha }({{x}}_{1},\cdot \cdot \cdot ,{{x}}_{n})=(-{\mathrm{1)}}^{\bar{\sigma }}\sum _{\alpha \mathrm{=1}}^{n+1}\prod _{i\mathrm{=1}}^{n}{a}_{\alpha }({{x}}_{i}){b}_{\alpha }({{x}}_{n+1}){{\rm{\Psi }}}_{N=n}^{\alpha }({{x}}_{1},\cdot \cdot \cdot ,{{x}}_{n}),$$

then ${{\rm{\Psi }}}_{N=n+1}({{x}}_{1},\cdot \cdot \cdot ,{{x}}_{n+1})$ becomes an antisymmetric wave function and yields the PD just given by the left-hand side of Eq. (13). Therefore, if we use the expression Eq. (28), then we have to search a set of functions *a*_*α*_(*x*_*i*_) and *b*_*α*_(*x*_*n* + 1_) that satisfy Eqs. (29)–(31) instead of Eqs. (11) and (12) in the above-mentioned concrete steps.

Similarly to the above, we can choose the other expression for ${{\rm{\Psi }}}_{N=n+1}({{x}}_{1},\cdot \cdot \cdot ,{{x}}_{n+1})$ instead of Eq. (14) or (28). For example, ${{\rm{\Psi }}}_{N=n+1}({{x}}_{1},\cdot \cdot \cdot ,{{x}}_{n+1})$ is supposed to be expressed by using ${{\rm{\Phi }}}_{SSD}^{\alpha }({{x}}_{1},\cdot \cdot \cdot ,{{x}}_{n})$ that are constructed by using given spin orbitals *ϕ*_*λ*_(*x*), ${\varphi }_{\mu }({x}),\cdot \cdot \cdot$, and yields ${\gamma }_{SSD}^{\mathrm{(2)}}{({\bf{rr}}^{\prime} ;{\bf{rr}}^{\prime} )}_{N=n}^{ijk\cdot \cdot \cdot \alpha }$, i.e., we suppose

32 $${{\rm{\Psi }}}_{N=n+1}({{x}}_{1},\cdot \cdot \cdot ,{{x}}_{n+1})=\sum _{\alpha \mathrm{=1}}^{n+1}\prod _{i\mathrm{=1}}^{n}{a}_{\alpha }({{x}}_{i},{{x}}_{n+1}){{\rm{\Phi }}}_{SSD}^{\alpha }({{x}}_{1},\cdot \cdot \cdot ,{{x}}_{n}),$$

where *a*_*α*_(*x*_*i*_, *x*_*n* + 1_) denote a function that should be determined. If *a*_*α*_ (*x*_*i*_, *x*_*n* + 1_) satisfies the following conditions;

33 $$\int \prod _{i\mathrm{=1}}^{n}{a}_{\alpha \text{'}}{({{x}}_{i},{{x}}_{n+1})}^{\ast }{a}_{\alpha }({{x}}_{i},{{x}}_{n+1})d{x}_{n+1}={\delta }_{\alpha ,\alpha \text{'}}\frac{1}{n+1}\frac{{|{{\rm{\Psi }}}_{N=n}^{\alpha }({{x}}_{1},\cdot \cdot \cdot ,{{x}}_{n})|}^{2}}{{|{{\rm{\Phi }}}_{SSD}^{\alpha }({{x}}_{1},\cdot \cdot \cdot ,{{x}}_{n})|}^{2}},$$

34 $$\sigma \sum _{\alpha \mathrm{=1}}^{n+1}\prod _{i,j\mathrm{=1}}^{n}{a}_{\alpha }({{x}}_{i},{{x}}_{n+1}){{\rm{\Phi }}}_{SSD}^{\alpha }({{x}}_{1},\cdot \cdot \cdot ,{{x}}_{n})={(-\mathrm{1)}}^{\bar{\sigma }}\prod _{i,j\mathrm{=1}}^{n}{a}_{\alpha }({{x}}_{i},{{x}}_{n+1}){{\rm{\Phi }}}_{SSD}^{\alpha }({{x}}_{1},\cdot \cdot \cdot ,{{x}}_{n}),$$

then Eq. (32) becomes an antisymmetric (n + 1)-electrons wave function and yields the PD just given by the left-hand side of Eq. (13). Therefore, if we use the expression Eq. (32), then what we have to do in the concrete steps is to search a set of functions *a*_*α*_(*x*_*i*_, *x*_*n*+1_) that satisfy Eqs (33) and (34) instead of searching a set of functions that satisfy Eqs (11) and (12).

Thus, various expressions for ${{\rm{\Psi }}}_{N=n+1}({{x}}_{1},\cdot \cdot \cdot ,{{x}}_{n+1})$ can be adopted. This means that the present method can provide various prescriptions to construct the *N*-representable LO-Jastrow PD.

## Concluding remarks

In this paper, the sufficient conditions for the *N*-representability of the LO-Jastrow PD are discussed. Using the properties of the LO-Jastrow PD, we derive the sufficient conditions that are imposed on the correlation function of the Jastrow wave function. As shown in the previous section, additional steps to search the suitable correlation function, which satisfies the sufficient conditions, are attached to the computational scheme proposed previously^17^. Although the number of steps rapidly increases with that of electrons, the concrete steps that are presented in the previous section are feasible for a small-electron system. Of course, there is a possibility that the suitable correlation function cannot be found out. In this case, as mention in the previous section, we can adopt other expressions for ${{\rm{\Psi }}}_{N=n+1}({{x}}_{1},\cdot \cdot \cdot ,{{x}}_{n+1})$, so that we may possibly find out a suitable correlation function. Otherwise, as mentioned in the previous paper, LO-Jastrow PDs are approximately *N*-representable in a sense that they satisfy four kinds of necessary conditions^17, 42–47^.
